# Surface Structure Analysis of Initial High-Temperature Oxidation of SS441 Stainless Steel

**DOI:** 10.3390/ma14206136

**Published:** 2021-10-15

**Authors:** Tung-Yuan Yung, Hui-Ping Tseng, Wen-Feng Lu, Kun-Chao Tsai, Tien Shen, Hsin-Ming Cheng, Jeng-Shiung Chen, Po-Tuan Chen

**Affiliations:** 1 Nuclear Fuels and Materials Division, Institute of Nuclear Energy Research, Taoyuan 325, Taiwan; romeoyung@yahoo.com (T.-Y.Y.); huiping@iner.gov.tw (H.-P.T.); wflu@iner.gov.tw (W.-F.L.); tsaijohn@iner.gov.tw (K.-C.T.); shentien@iner.gov.tw (T.S.); 2 Department of Electronic Engineering and Organic Electronics Research Center, Ming Chi University of Technology, New Taipei City 243, Taiwan; 3 Yottadeft Optical-Electron Company, Taipei City 104, Taiwan; jsc@yottadeft.com; 4 Department of Vehicle Engineering, National Taipei University of Technology, Taipei 106, Taiwan

**Keywords:** solid oxide fuel cells, interconnect, high temperature, spinel, oxidation

## Abstract

Chromia-forming ferritic stainless steel (FSS) is a highly promising interconnect material for application in solid oxide fuel cells. In this study, initial oxidation of chromium oxides was performed at 500–800 °C to understand the evolution of materials at an early stage. The structural variations in oxide scales were analyzed through scanning electron microscopy, energy dispersive spectroscopy (EDS), transmission electron microscopy (TEM), X-ray diffractometry (XRD), laser confocal microscopy (LSCM), X-ray photoelectron spectroscopy (XPS), and Raman spectroscopy. Surface electrochemical properties were investigated through electrochemical impedance spectroscopy to understand how the heat treatment temperature affected surface impedance. Treatment temperatures higher than 700 °C facilitate the diffusion of Cr and Mn, thus allowing ferritic spinels to form on the surface and leading to high electrical conductivity.

## 1. Introduction

Among currently available fuel cell types, solid oxide fuel cells (SOFCs) are preferred for stationary power generation and auxiliary power unit applications because they demonstrate high efficiency and fuel flexibility [1,2,3,4]. In stacks of SOFCs, individual cells are separated by interconnects, which provide the flow field pattern for the anode and cathode gases and are electrically connected throughout the stack [5]. Ferritic stainless steel (FSS) is the material most frequently chosen for SOFCs because of its impressive machinability, low cost, easy processing and molding, and thermal expansion coefficient similar to other SOFC components [6,7]. However, at high working temperatures (500–800 °C) and after long-term discharge operation, the Fe on the FSS surface forms harmful oxide layers, resulting in increases in resistance, spallation on the material surface, and a decline in fuel cell performance [8,9,10].

FSS alloys commonly contain 17–24 wt% chromium (Cr). Prior formation of Cr oxide layers on the metallic interconnect surface can suppress the Fe oxide generated there and protect the alloys from corrosion [11,12]. Passive Cr oxide layers automatically grow on the surface of FSS, even before SOFC operation, because of the high Cr content in the ambient atmosphere. A moderately thick chromium oxide layer has satisfactory electrical conductivity; thus, the chromium oxide scale must be maintained at below a certain thickness so as to maintain a low electrical resistance over the interconnects [13,14]. Cr oxide of a certain thickness exhibits beneficial long-term oxidation resistance, but Fe oxides are less protective and grow more rapidly than Cr oxides. In addition, Cr oxides provide effective adherence to metal substrates and do not spall off with certain coating parameters [15].

Another common problem with SOFCs is that the high flow rate of ambient air on the cathode side causes the evaporation of volatile Cr species, which reduces the catalytic activity of the cathode [16,17,18]. Cr evaporation leads to the consumption of Cr from the steel interconnect. Consequently, Fe is oxidized rapidly because of the reduced protection by chromium oxides, a process commonly referred to as breakaway oxidation [19,20]. Therefore, an appropriate Cr oxide layer on the surface of FSS can inhibit element volatilization by acting as a diffusion barrier [21].

The corrosion stability of FSS therefore depends on the formation of Cr oxide layers. To form a continuous protective chromium oxide layer, the supply or flux of Cr from the bulk alloy must compare sufficiently with Cr consumption through oxidation at the oxide–metal interface [22]. The temperature adjustment during initial Cr oxidation is a crucial parameter [23,24]. Several studies have performed oxidation at temperatures of 800–900 °C [25]. However, SOFC research is targeting lower operating temperatures to decelerate the degradation of cell components. An operating temperature of approximately 600 °C is sufficient to maintain satisfactory ionic conductivity of the electrolyte [26]. Although metal oxidation rates are expected to decrease at lower temperatures, several FSS studies have demonstrated that, depending on the atmosphere and alloy composition, the oxidation rate can increase when the temperature is reduced within certain intervals [27,28,29]. Moreover, Young et al. studied several FSSs at temperatures ranging from 500–900 °C and revealed that oxidation was generally more severe in the 500–650 °C region than in the 650–800 °C region, because more iron oxide nodules formed at the lower temperatures [30]. Goebel et al. reported a dual atmosphere in a pre-oxidation process at 800 °C; the oxide scales contained Fe-rich nodules and a Cr oxide layer for corrosion resistance [31].

Morphological variations in the short operation time of the initial oxidation have rarely been discussed. However, analyzing the variations of alloys during a short oxidation period can clarify how each element diffuses in an alloy. Moreover, during the initial oxidation stage, the effects of morphological variations are significant.

A commercially available FSS called AISI SS441 was chosen as the test material because it is less expensive than Crofer 22 APU, Crofer 22H, Sanergy HT, and ZMG 232. After the initial protective oxidation, interconnectors have three primary requirements: (i) reliable electrical conductivity; (ii) chemical stability under high-temperature oxidation and in a reducing atmosphere; and (iii) effective oxidation, vulcanization, and carbonization resistance.

Herein, we discuss the microstructural features formed after 10-min oxidation at initial temperatures of 500–800 °C. The morphologies of surface oxide scales and the elemental distributions were investigated through scanning electron microscopy (SEM) and energy-dispersive spectroscopy (EDS). The nanoscale regions of the oxides were studied through transmission electron microscopy (TEM), XRD, and Raman spectroscopy. Electrochemical impedance spectroscopy (EIS) was used for comparing the chemical impedance of the SS441 pre-oxidized at different initial temperatures and for simulating equivalent circuits.

## 2. Experimental Methods

### 2.1. Heat Treatment

The chemical composition of AISI SS441 is presented in Table 1. SS441 specimens were cut into 10 × 10-mm^2^ pieces with a thickness of 2 mm, and their surfaces were ground using 1200-grit sandpaper. The specimens were placed exposed in a furnace at 500, 600, 700, or 800 °C for 10 min and heated at a rate of 5 °C/min (Eurotherm 2416 program controller) from room temperature (approximately 27 °C), and dried in air at a flow rate of 2 L/min; thereafter, the samples were cooled at 15 °C/min without air flow.

### 2.2. Surface Morphology

After heat treatment, the oxide scales were examined through surface electron microscopy to determine their morphologies, elemental compositions, and phases. A JOEL JEM 7600 surface electron microscope coupled with an Oxford Max energy-dispersive spectroscope without liquid nitrogen cooling, and a JOEL FE2100F transmission electron microscope were used to investigate the electron diffraction patterns of the oxide scales after pretreatment at different temperatures. In this study, the samples were prepared through focused ion beam milling and an in situ lift-out technique using an FEI Versa 3D Dual Beam instrument (FEI Company, Hillsboro, Oregon, OR, USA).

### 2.3. Microscale Surface Roughness

The microscopic surface roughness and cross-sections of the samples were examined using an LEXT OLS 4000 laser confocal microscope (Olympus Corporation, Tokyo, Japan).

### 2.4. Oxide Scale Composition and Raman Spectra

The Oxford Max energy-dispersive spectroscope and a Bruker D8 Discover X-ray diffractometer with Cu Kα radiation (λ = 1.5406 Å) were used to analyze the elemental composition and crystalline phases of the oxide scales at a scanning rate of 4°/min over a 2 θ range of 15°–80° with an incident angle of 0.5°. A B&W Tek iRaman Plus spectrometer was used to analyze all samples with a 785-nm laser and a 600-mm slit width. X-ray photoelectron spectroscopy (XPS) was performed on a Thermo VG ESCALAB 250 equipped with a dual anode (Mg Ka/Al Ka) X-ray source.

### 2.5. Surface Impedance

An Autolab PASTAT 30 (AUTOLAB, Utrecht, Netherlands) was used to examine the oxide scales in a three-electrode cell with a Pt wire as the counter electrode, Ag/AgCl as the reference electrode, and the heat-treated specimens welded to a Pt wire as the working electrode. The electrolyte was 0.1 M NaCl (aqueous) at room temperature. EIS was performed with an open-circuit potential amplitude of 10 mV. EIS equivalent circuit analysis was performed on Zview. EIS is a powerful method for quantifying the three parameters defining a corrosion process: (i) the corrosion rate, through the charge transfer resistance (R_ct_) (W cm^2^) and using Faraday’s law to estimate the penetration of the attack (μm/day); (ii) diffusion processes defined by the parameter Warburg (W cm^2^/s^1/2^); and (iii) electrochemical double-layer capacitance at the metal–solution interface (C_dl_) (F/cm^2^).

## 3. Results

### 3.1. Oxide Scale Morphology and Elemental Analysis

**Figure 1:**
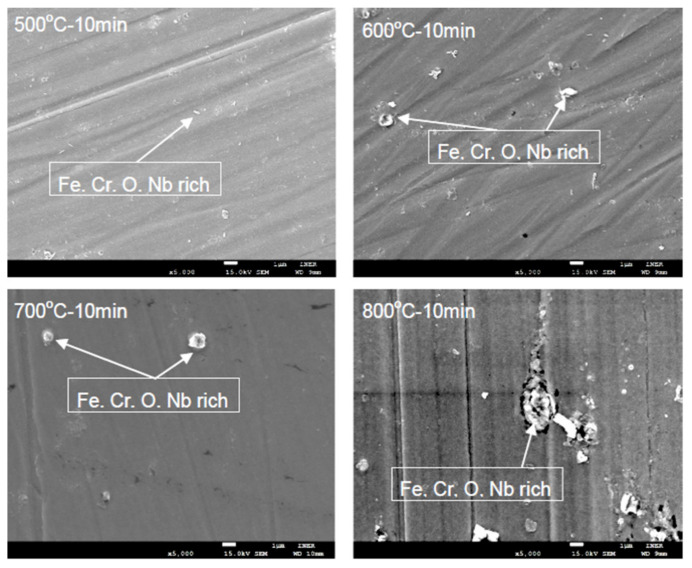
Surface morphology after treatment at 500, 600, 700, and 800 °C for 10 min (scale bar: 1 μm).

Figure 1 presents the surface morphology of the oxide scales after heat treatment at 500, 600, 700, and 800 °C. The Fe, Cr, O, and Nb oxides glowed up as the temperature increased. The particle-like oxides were noted in the tool scratch line. The surface defects also enhanced oxidation at higher temperatures.

The elemental compositions were analyzed through energy dispersive spectroscopy (EDS), and the results are listed in Table 2. The Cr content was the highest at 800 °C (17.41%) and the lowest at 700 °C (14.72%). As shown in Table 1, the Cr content in the specimen as received was 17.70%. Cr evaporation pressure was lower than that of Fe, Mn, and Nb. Chromium oxide formed the protection layer on the FSSs. Nevertheless, the Fe and Nb contents were the same as Cr after accounting for differences in heat treatments. Furthermore, it was revealed that the oxygen content in the oxide scale compositions increased with increasing heat-treatment temperatures. At 800 °C, the oxides were spread more widely than at other temperatures. However, manganese was not detected through EDS for specimens treated at temperatures below 800 °C.

Laser confocal microscopy with z-axis autofocusing revealed the surface roughness of the SS441 samples after heat treatment at various temperatures. The surface roughness was 20.35 ± 4.23, 30.55 ± 7.88, 37.27 ± 7.88, and 47.54 ± 9.87 nm for the treatment temperatures of 500, 600, 700, and 800 °C, respectively.

**Figure 2:**
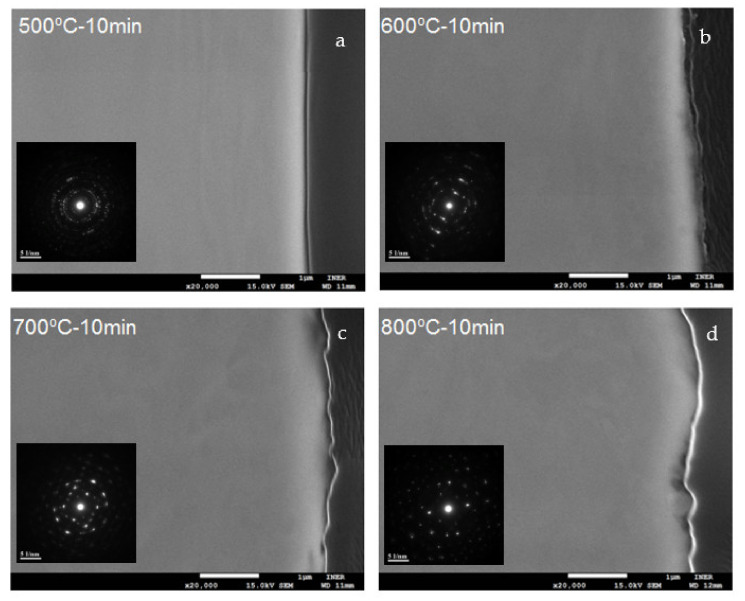
Cross-sectional SEM and TEM images of samples treated at (**a**) 500, (**b**) 600, (**c**) 700, and (**d**) 800 °C for 10 min (scale bar: 1 μm). The insets in the bottom left are the selected area diffraction patterns, with scale bars of 0.05 nm.

Cross-sectional images of the heat-treated SS441 specimens are displayed in Figure 2. The oxide scale surface revealed increasing curvatures at higher heat-treatment temperatures, coherent with the surface roughness profiles. When the energy input was increased, the elements increasingly evaporated at higher temperatures. Thus, the surface roughness and curvature were observable in the cross-sectional images.

**Figure 3:**
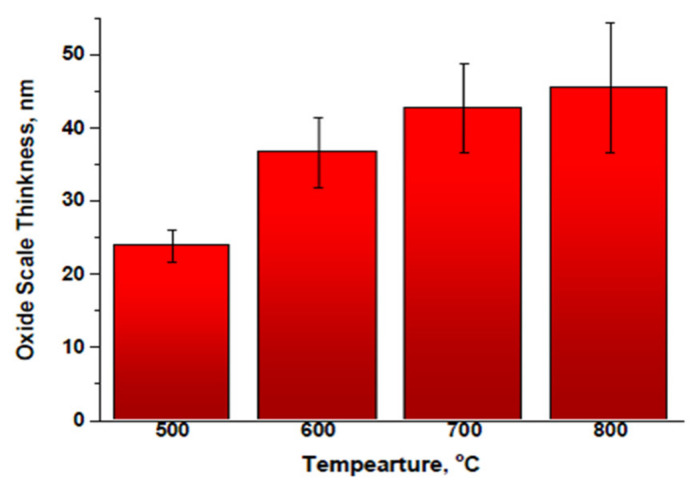
Oxide scale thicknesses as determined from cross-sectional SEM images.

The oxide scale cross-sectional thicknesses were calculated with the JOEL JEM 7600 and are presented in Figure 3. The thickness differences decreased with increasing heat-treatment temperatures. The approximate thickness differences were 13.2, 12.0, and 3.1 nm at 500–600, 600–700, and 700–800 °C, respectively. Thus, oxide scale propagation was most favorable in the region of 500–700 °C. Additionally, the oxide scale surface roughness increased with the pre-oxidation temperature. Elemental diffusion from the substrate was stronger at 800 °C because a Cr oxide scale had formed.

The cross-sectional images in Figure 2 display the oxide scales, which exhibit the second Cr oxide layer, or spinel structures, on the top of the surface. However, identification of the specific crystalline phase was difficult. Clear ring patterns formed at temperatures of 500–700 °C. The oxide scale formed at 800 °C revealed only a partial ring pattern. The Cr oxide scales transitioned into a spinel crystal structure, as determined through the SAED analysis method for SS441 with the pre-oxidation at 800 °C for 10 min. At heat treatment temperatures of 500 and 600 °C, the electron diffraction ring patterns indicated the rearrangement of the crystal structures of the oxides.

Furthermore, at higher temperatures, the surface roughness was altered by the spinel crystal formation. The low-melting-point elements possibly evaporated because of the higher kinetic energy produced by the higher temperatures. The oxide scales were investigated through TEM, and the results are shown in Figure 3. Each bar was 25 measurements on average, with the error ±0.7 nm, ±1.6 nm, ±2.1 nm, and ±3.4 nm for 500 °C, 600 °C, 700 °C, and 800 °C, respectively.

### 3.2. Oxide Scale Crystalline Size and Chemical Environment

**Figure 4:**
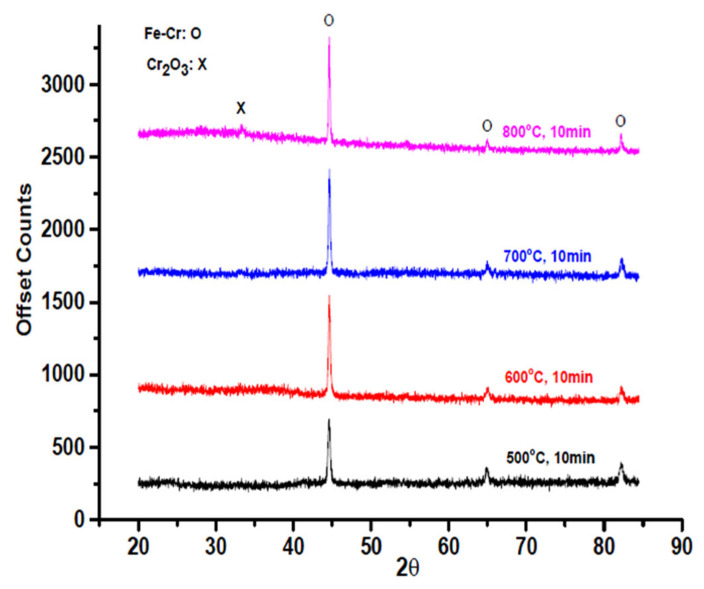
Oxide scale thicknesses as revealed in cross-sectional analysis through transmission electron microscopy.

Oxide scale propagation after heat treatment was notable with regard to the crystalline phase. XRD revealed the crystalline phases of the oxide scales, which the X-ray penetrated only to approximately 10 μm of depth. The XRD results are presented in Figure 4. The interconnect alloy contained a considerable amount of Cr and formed Cr_2_O_3_ scales to provide protection under the elevated temperatures. Mn was added to limit Cr diffusion and to lower the electrical contact resistance. Si was added for the suppression of the inverse effect of Nb in oxidation. Laves phase are formed in the substrate after heat treatment for FSS.

A 2θ Cr oxide peak was observed at 33.1° for the sample treated at 800 °C. The 2θ Fe–Cr peaks were at approximately 44°, 64.8°, and 82.5°. The spinel species in oxidized FSSs have received much attention, particularly when developed through the thermal conversion approach, due to their effectiveness in suppressing the growth of chromium oxide layers and reducing outward migration of Cr [32,33]. A grazing-incidence XRD (GIXRD) angle of 1° was used for the thin oxide scales. However, the 2 θ peaks at approximately 64.8° and 82.5° disappeared in the GIXRD analysis. The peaks at approximately 44° indicated the preferred orientation of the SS441 Fe–Cr structure. The substrate crystalline structure still dominated the diffraction peaks as the preferred orientation. However, different heat treatment temperatures and times can affect the crystalline size and orientation. The Scherrer equation for the peak width of the half peak high, revealing crystalline size, is as follows:

τ = κλ/FWHM cosθ(1)

where τ is the crystalline size, κ is a dimensionless shape factor with a value close to unity, λ is the X-ray wavelength, FWHM is the full width at half maximum, and θ is found in the x-axis.

The SS441 substrate may have contributed to the peak intensities shown in Figure 4, but the full width at half maximum changed with the temperature. As the temperature rises, the Cr diffusion rate increases. The Fr–Cr peaks for the pre-oxidation process indicated that the surface oxide structure served as a precursor for the spinel structure (Fe, Cr, Mn)_3_O_4_.

As shown in Table 3, the crystalline size increased with increasing temperature. The spinel crystalline sizes were 31.80, 39.84, 41.71, and 47.79 nm at 500, 600, 700, and 800 °C, respectively. The initial oxidation required a short time at the various temperatures. Furthermore, the peak ratios indicated the presence of crystalline structures.

The Bruker EVA software program uses a semi-quantitative method for analyzing the crystalline composition; the results of the analysis are presented in Table 4. At 600 °C, the α-Fe crystalline content was the lowest (9.2%), and the Fe–Cr crystalline was the highest (69.3%). Spinel crystallization occurred at approximately 600 °C. Treatments at higher temperatures revealed no considerable differences in their crystal structures. For long-term SOFC application, an operational temperature below 600 °C should thus be considered.

**Figure 5:**
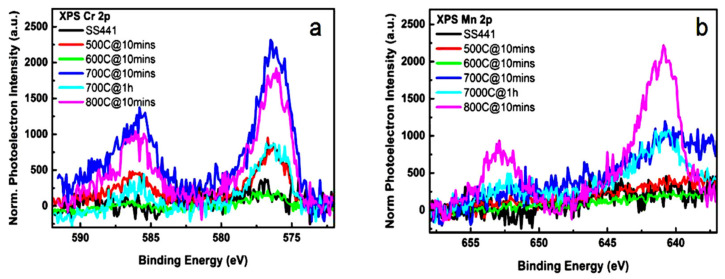
XPS results for oxide scales of specimens pre-oxidized at 500, 600, 700, and 800 °C. (**a**) Cr 2p (**b**) Mn 2p.

The oxide scale chemical environment was investigated through XPS. The Cr 2p and Mn 2p are presented in Figure 5. The highest Cr 2p and Mn 2p peak intensities were at 700 and 800 °C, respectively. The highest Cr and Mn diffusion rates were observed at 700 and 800 °C, respectively. However, no notable peaks appeared at 600 °C. The cross-sectional SEM/EDS data in Table 2 reveal Mn content of 0.4 wt% for pre-oxidation at 700 °C-10 min. The Mn 2p peak was apparent after heat treatment at 700 °C. Heat treatment for 1 h at 700 °C resulted in a decrease in the Cr 2p peak intensity but an increase in the Mn 2p peak intensity. Thus, 700 °C is the critical temperature for the Cr/Mn ratio of the oxide scales. Using a curve fitting analysis for the Cr 2p and Mn 2p spectra, the ratios of CrO_2_/CrO_3_ were found to be 2/11, 50/19, 2/7, and 25/6 at 500, 600, 700, and 800 °C, respectively. Before using metallic interconnects for SOFC, the aforementioned factors should be considered. The higher the Mn content, the greater the electrical conductivity. Spinel minerals belong to a large group of compounds with cubic symmetry (space group Fd3m, Hermann–Mauguin notation Oh); the general chemical formula AB_2_X_4_, where A and B are cations with variable valences and X is an anion, which can be O_2_^−^, S_2_^−^, Se_2_^−^, or Te_2_^−^ [34,35].

**Figure 6:**
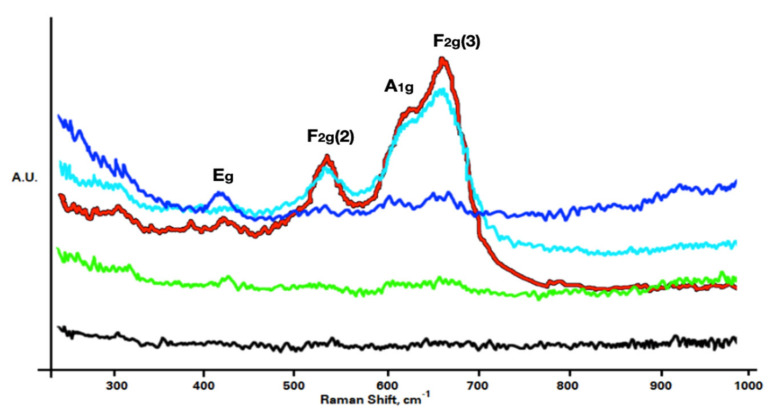
Raman spectra at 785 nm revealing vibrational modes for oxide scales. The black, green, blue, light blue, and red indicate the as-received, 500, 600, 700, and 800 °C samples, respectively.

Raman spectroscopy is widely used for the routine identification of materials and has highly promising applications in spinel materials. It may aid in distinguishing spinel species from characteristic spectral patterns (“fingerprints”) without preliminary information about the composition and structural origin of individual features. D’lppolito et al. reported the Raman spectra of chromate, aluminate, and ferrite spinels in 2015 [35]. The ferrite spinels they used for Raman analysis were FeFe_2_O_4_ and FeCr_2_O_4_. The authors revealed four peaks for F_2_g(1), Eg, F_2_g(2), and F_2_g(3). The FeFe_2_O_4_ spinel was revealed by the A_1_g vibrational mode at approximately 675 cm^−1^. They also reported three peaks for the MnAl_2_O_3_ spinel at 200, 400, and 520 cm^−1^ for F_2_g(1), Eg, and F_2_g(2), respectively. The F_2_g(2) peak of MnAl_2_O_4_ and A_1_g peak of FeFe_2_O_4_ are visible in the Raman spectra at 600–750 cm^−1^. The laser wavelength for the Raman analysis was 785 nm; in Figure 6, the red and green spectra reveal that heat treatment below 700 °C did not exhibit the Eg, F_2_g(2), and F_2_g(3) peaks at 418, 531, and 681 cm^−1^, respectively. A1g shoulder peaks were observed at a wavenumber of 623 cm^−1^. These A1g peaks appeared with temperatures above 600 °C. The three Raman-active F2g modes are labeled as F2g(1), F2g(2), and F2g(3), with F2g(1) being associated with the lowest Raman shift and F2g(3) indicating the Raman F2g symmetry mode at the highest wavenumber. However, above 600 °C, the F2g(3) peaks for the SS441 specimens included shoulder peaks at approximately 623 cm^−1^, indicating the presence of a ferritic spinel structure with Mn, Fe(Cr,Mn)_2_O_4_. Moreover, the F_2_g(3)/A_1_g ratio was approximately 3, effectively the same as the Fe–Cr/Fe_19_Mn ratio in Table 4.

### 3.3. Electrochemical Properties of Oxide Scales

**Figure 7:**
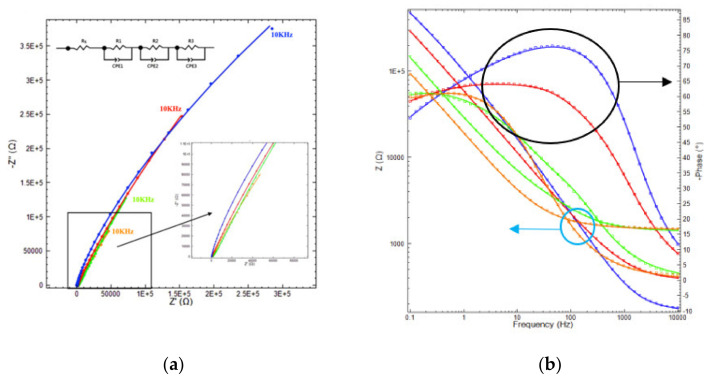
Nyquist (**a**) and Bode (**b**) and plots for heat treatments at 500 °C, 600 °C, 700 °C, and 800 °C with blue, red, green and orange, respectively. The dots are EIS data, and the line is the simulated equivalent circuit.

After heat treatment at various temperatures for 10 min, the electrochemical properties of the oxide scales were analyzed. The EIS equivalent circuit was analyzed using Zview software; the results revealed that increasing heat-treatment temperatures directly increased electrochemical impedance. The EIS equivalent circuit fitting results are presented in Figure 7 and Table 5. The EIS equivalent circuit fitting scheme is displayed in Figure 7.

Two equivalent circuits were employed for SS41 heat treatments at ≥600 °C. One was R(QR)(QR), and the other was R(QR)(QR)(QR); here, R is electrolyte resistance, and (QR) is the electrical resistance and constant phase element (CPE) of the oxide layer on the SS441 surface. Moreover, R is defined by the electrical resistance of charge transfer. The trend increases with increasing temperature. CPE could be the capacity between the different oxide layers. The third (QR) noted after heat treatment at 700 °C indicated the highest resistance.

The Nyquist plots for 10 kHz from the highest resistance to the lowest resistance are shown on the right side of Figure 7. The phase angles and real resistances in Bode plot show a decline in the response frequency of the resistance turning point as the phase angle shifted with the heat treatment temperature from 500 to 800 °C, as also shown on the right side of Figure 7. The ratio of Fe–Cr to Fe_19_Mn (Table 4) determined through semiquantitative XRD decreased as the treatment temperature increased, meaning the Mn element diffused more from the substrate to the surface.

In our previous study on the pre-oxidation of SS441 for 25 h, the surface spinel structure (Mn,Cr)_3_O_4_ was observed from a top view and through cross-sectional SEM/EDS analysis [36]. Nevertheless, the 10-min pre-oxidation of SS441 did not produce (Mn,Cr)_3_O_4,_ but did produce (Fe,Cr,Mn)_3_O_4_ instead. The Rs value and oxide scale thickness increased with the heat treatment temperature, as shown in Table 5.

R1, R2, and R3 are defined respectively as the electron–metal resistance, the oxide–oxide resistance, and the oxide–substrate resistance. The values of R1 for the samples preoxidized at 700 and 800 °C were 1.02 and 2.51 kΩ, respectively. The highest values for R2 and R3 were for the sample treated at 600 °C. The capacitance also rose with the pre-oxidation temperature. The Mn diffusion from the substrate to the surface enhanced the electrical conductivity of the oxide scales and enabled the electron double-layer to serve as a capacitor. The XPS spectra in Figure 5 reveal that the Mn diffused at pre-oxidation temperatures above 600 °C. The SEM/EDS results also revealed Mn diffusion from the substrate to the surface for temperatures above 600 °C. Thus, the Mn content in the oxide scale enhanced the electrical conductivity after pre-oxidation of SS441 at such temperatures.

## 4. Conclusions

The pre-oxidation of SS441 interconnects for SOFC can improve fuel cell performance by enhancing electrical properties. The structural variations and electrochemical behavior of interconnects with initial oxidation at different temperatures has been analyzed systematically. The experiments in the present study revealed that heat treatment of FSS at approximately 700 °C, at which Cr oxides transition into (Fe, Cr, Mn)_3_O_4_, enhanced electrical conductivity. As revealed by the XRD and Raman spectra, the oxide scales were ferritic spinel (Fe, Cr, Mn)_3_O_4_. The Cr and Mn content in the oxide scales after heat treatment increased with the treatment temperature. In EDS, XRD, XPS, Raman, and EIS analysis, Mn content was found in the oxide scales of only those samples treated at temperatures above 700 °C. EIS revealed the presence of three (QR) layers in the treated samples. The temperature of 600 °C for 10-min pre-oxidation is the threshold for Mn evaporation. A combination of a pre-oxidation process and a protective coating layer should be investigated in the future.

## Figures and Tables

**Table 1:** Elemental composition of SS441.

Element	Cr	Mn	Nb	C	Si	Ti	S	Fe
Wt%	17.7	0.30	0.37	0.015	0.55	0.15	0.002	Bal.

**Table 2:** Elemental composition determined through EDS.

Atomic %	500 °C-10 min	600 °C-10 min	700 °C-10 min	800 °C-10 min
Fe	63.99	63.69	62.85	69.10
Cr	16.01	15.92	14.7	17.41
Nb	0.39	0.27	0.23	0.39
Mn	-	-	0.40	0.45
O	5.71	6.34	7.7	11.46

**Table 3:** XRD peak analysis with Scherrer’s equation.

Entry	Peak Position (2 θ)	FWHM	Size (nm)
SS441	44.65°	0.3324°	25.12
500 °C-10 min	44.61°	0.3601°	31.80
600 °C-10 min	44.65°	0.2951°	39.84
700 °C-10 min	44.65°	0.2827°	41.71
800 °C-10 min	44.64°	0.2441°	47.79

**Table 4:** Crystalline compositions of SS441 surfaces using the semiquantitative method and Bruker EVA XRD analysis software.

	Fe	Fe-Cr	Fe_19_Mn
500 °C, 10 min	48.4%	39.0%	12.5%
600 °C, 10 min	9.2%	69.3%	21.5%
700 °C, 10 min	15.8%	62.9%	21.3%
800 °C, 10 min	16.6%	60.1%	23.3%

**Table 5:** Results of equivalent circuit simulation.

	Rs (kΩ)	R1 (kΩ)	Capacitor (mF)	R2 (MΩ)	CPE1/n (μMho)	R3 (kΩ)	CPE2/n (μMho)
500 °C-10 min	0.17	4.58	0.794	2.96	2.25/0.581	-	-
600 °C-10 min	0.37	9.18	1.01	4.56	3.83/0.636	1.26	8.02/0.703
700 °C-10 min	1.39	1.02	2.23	0.73	14.8/0.784	2.36	21.9/0.589
800 °C-10 min	1.46	2.51	4.45	1.10	11.3/0.589	2.05	76.6/0.526

## Data Availability

Data sharing is not applicable for this article.
